# Osmotic Demyelination Syndrome in a Patient with Hypokalemia but No Hyponatremia

**DOI:** 10.1155/2020/3618763

**Published:** 2020-03-23

**Authors:** Carolina Ormonde, Raquel Cabral, Sara Serpa

**Affiliations:** ^1^Nephrology Department, Hospital do Divino Espírito Santo, Ponta Delgada, Portugal; ^2^Radiology Department, Hospital do Divino Espírito Santo, Ponta Delgada, Portugal

## Abstract

Osmotic demyelination syndrome (ODS) is characterized by loss of myelin in various parts of the central nervous system. It is mainly caused by a rapid correction of hyponatremia, although other factors that may cause rapid rise in serum osmolality can also be associated with its development. Its prognosis is poor and the recovery rate is unknown. The authors report a rare case of a patient with multiple risk factors for ODS, without hyponatremia, who developed ODS and surprisingly recovered. This case report highlights the importance of recognizing risk factors for the development of ODS, even if the main one is not present.

## 1. Introduction

Osmotic demyelination syndrome (ODS) is a rare condition [1] characterized by loss of myelin in various parts of the central nervous system. It is subdivided into central pontine myelinolysis (CPM) and extrapontine myelinolysis (EPM), depending on what level the demyelination occurs [2]. The main risk factor consists in a rapid correction of chronic hyponatremia [3], but rare cases of ODS without the latter have been reported. Other known risk factors for ODS are malnutrition, alcoholism, hypokalemia, use of diuretics, and fluid resuscitation [2]. Symptoms may range from confusion to coma and can frequently be delayed some days after the trigger event [4]. Magnetic resonance imaging (MRI) is the key method for diagnosis, and its treatment is mainly supportive [5]. The overall prognosis seems to be poor, and the recovery rate is unknown [6]. We report a case of a patient who, unexpectedly, developed ODS without evidence of hyponatremia but also had multiple risk factors for its development.

## 2. Case Presentation

A 55-year-old male with a history of insulin dependent type 2 diabetes mellitus was admitted to the nephrology department with anasarca. He had a nephrotic syndrome for at least 2 years caused by diabetic nephropathy and had history of nonadherence to medication. Despite the generalized edema, he was noticeably malnourished. The patient was treated with high doses of furosemide and was also given ceftriaxone for a urinary tract infection. After eight days of hospital admission, he developed hypovolemic shock caused by a pseudomembranous colitis due to *Clostridium difficile*, for which aggressive fluid resuscitation was needed. He was given antibiotics for the pseudomembranous colitis—4 days of vancomycin to which he did not respond, and he was then switched to fidaxomicin. He also developed hypokalemia which was corrected with intravenous potassium chloride (60 meq a day for 5 days). Additionally, he had poor glycemic control for which insulin was instituted. Six days after this complication, he developed a slurred speech and a progressive decline in his level of consciousness throughout the course of the next two days. Neurologic examination revealed quadriplegia and, few days later, he developed a “locked-in syndrome.”

**Figure 1:**
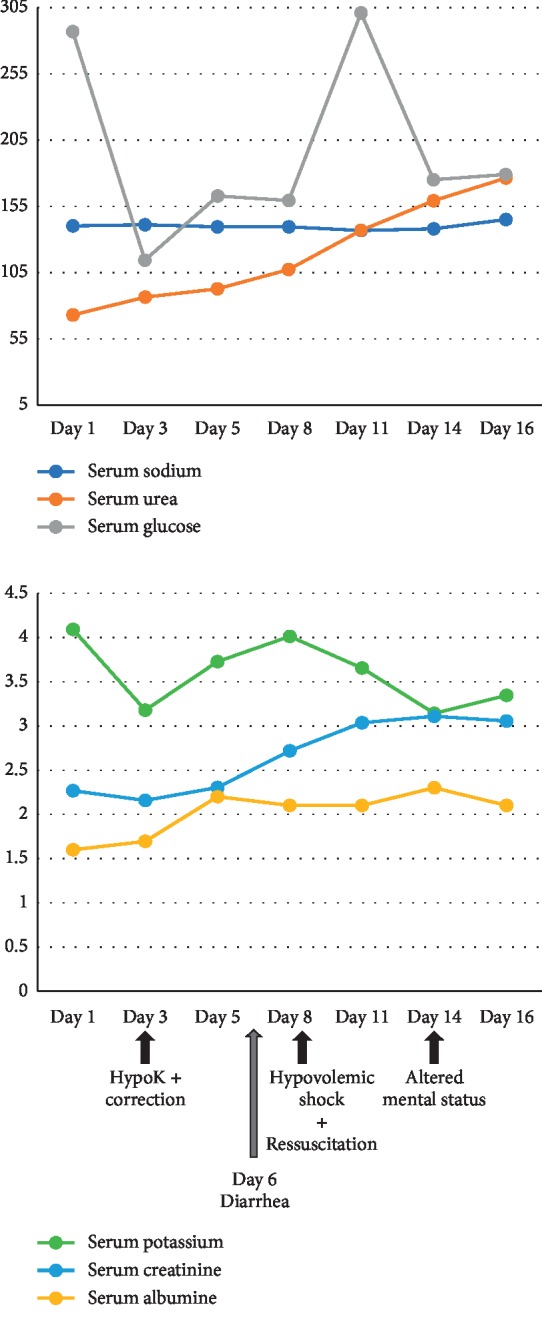
Correlation between sequence of events and serial laboratory results. Day 1 corresponds to the day of admission. Value units are the following: mg/dL for serum urea, creatinine, and glucose; mmol/L for serum sodium and potassium; g/dL for serum albumin.

**Figure 2:**
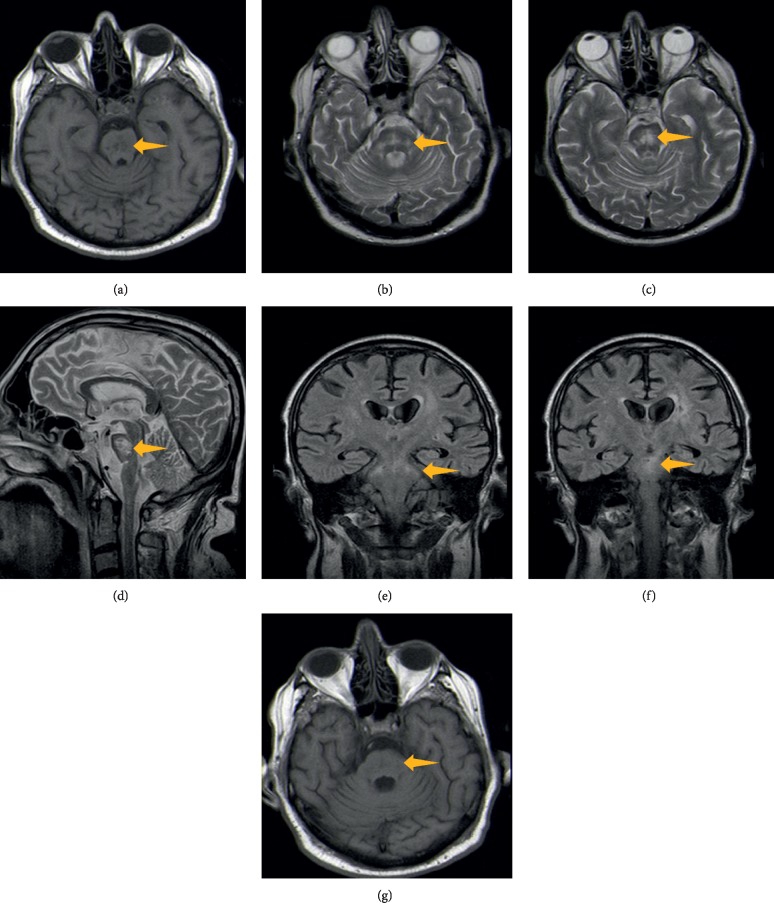
MRI images show central pontine T1 moderately hypointense areas (a), T2 and FLAIR areas of hyperintensity at the level of the pons (b, c, d, f), including an area trident-shaped of hyperintensity at the level of the pons on axial T2 (b) (typical appearances of the pons in osmotic demyelination syndrome), that extends to cerebellar peduncles (b, e); after gadolinium, there was no enhancement (g).

There were no electrolyte disturbances in blood analysis by the time the patient developed these symptoms (Figure 1). The only relevant alterations were his usual severe hypoalbuminemia and hyperglycemia. Brain computed tomography (CT) did not show acute lesions. Cerebrospinal fluid analysis showed 1 red blood cell/mm^3^, normal glucose and protein levels, negative Gram stain, Ziehl–Neelsen stain and bacterial culture, and negative PCR for Herpes simplex virus. Electroencephalogram revealed diffuse and symmetric slow wave activity. An MRI was then performed revealing heterogeneous T1-hypointense, T2-hyperintense, and FLAIR-hyperintense areas located in the pons, cerebellar peduncles (mainly in the middle cerebellar peduncles), which were compatible with ODS (Figure 2). MRI also showed millimetric lacunae infarcts in corona radiata bilaterally and left cerebellum.

Supportive treatment was given to the patient, and, unexpectedly, he started recovering from coma over the following week. During this first week, he progressively recovered his level of consciousness with an intelligible speech. He also developed, during the recovery course, a bacteremia by *Acinetobacter baumannii* which was resolved with meropenem. Throughout the first month of recovery, he had a continuous and persistent program of physiotherapy and partially regained his legs and arms movements. When he was discharged, he was able to stand on orthostatic position and walk with bilateral support. He continued the rehabilitation program to regain complete autonomy.

## 3. Discussion

Adams et al. were the first to describe pontine myelinolysis in 1959 [7]. Today, it is known as ODS, and it is subdivided into CPM (the most frequent form) and EPM. [2] Although ODS is a rare condition, its true incidence is unknown and often underdiagnosed [1].

The main risk factor for ODS is rapid correction of chronic hyponatremia, particularly when it is lower than 120 meq/L [3]. Cerebral cells defend themselves from edema caused by chronic hyponatremia by altering their osmolality with gain in electrolytes and organic osmolytes. When hyponatremia is corrected too quickly, cells cannot readapt fast enough to the higher osmolality and are at risk of lysis. Oligodendrocytes are the most affected cells [1]. Other risk factors that may contribute to osmotic demyelination are as follows: malnutrition, chronic alcoholism, primary adrenal insufficiency, prolonged use of diuretics, hypokalemia, hyperglycemia, fluid resuscitation, hemodialysis, and liver transplant [2]. There are rare reports of ODS cases with mild or no hyponatremia, which demonstrates that a combination of other risk factors besides hyponatremia may also lead to ODS [3].

Frequently, symptoms are delayed for two to six days after rapid correction of osmolality and it can present in various ways [4]. Asymptomatic cases have also been described [3]. Clinical presentation of ODS is typically sequential [8], and the most common symptoms are confusion, muscle weakness, quadriplegia, oculomotor abnormalities, dysphagia, dysarthria, locked-in syndrome, and progressive deterioration of consciousness [9].

CT assessment of the skull base can be difficult due to beam hardening artifact. The preferable diagnostic method is MRI. Findings might be delayed up to four weeks after the initial symptoms [2]. It reveals T1-hypointense, T2-hyperintense, and FLAIR-hyperintense signals mainly in the pons. Moreover, there can be a typical sign of osmotic demyelination syndrome—“the trident sign”—where the symmetrical high T2/FLAIR signal abnormality appears located in central pons. This reflects the prevalent involvement of the transverse pontine fibers and relative sparing of the descending corticospinal tract. It may also be seen T1-hypointense, T2-hyperintense, and FLAIR-hyperintense signal changes in the basal ganglia, thalamus, cerebellum, hippocampus, and cerebral cortex [5]. The earliest change is perceived on diffusion (diffusion-weighted (DWI) MRI) with restriction in the lower pons. This is apparent within 24 hours of the beginning of quadriplegia. On apparent diffusion coefficient (ADC) map, there is signal loss. Our exam was performed on 1 Tesla MRI without DWI capacity nor ADC map obtainable.

The most important therapy is prevention of rapid correction of hyponatremia, or in susceptible patients, prevention of rapid changes in plasma osmolality. Treatment is mainly supportive, but some case reports have shown that the use of intravenous immunoglobulin or plasmapheresis may be useful as they may remove myelotoxic substances from plasma [5].

Overall, ODS has a poor prognosis with a high mortality rate. Patients who survive may not recover if in a coma and irreversible sequelae may persist [6]. No clinical or radiological features predict the outcome [5]. A high level of suspicion for ODS is the most important factor for early diagnosis and better outcome. The level and time of recovery varies and are uncertain [4].

We reported an unusual case of CPM with no hyponatremia. After the unexpected diagnosis by MRI, we concluded that, even in the absence of the latter in our patient, the combination of multiple risk factors contributed to the development of ODS. First, he had a prolonged history of diabetes mellitus with nephrotic syndrome and poor glycemic control, which contributed to severe proteinuria and malnutrition. During the first days after admission, he was also given a high and prolonged course of diuretics to treat the anasarca. After the abrupt diarrhea that caused the hypovolemic shock and hypokalemia, he was given a large amount of fluids. From our point of view, this was the main trigger event that caused the rapid change in osmotic balance. After this event, the chronological presentation of neurologic manifestations is consistent with ODS.

There are rare, but similar, cases reported of ODS without hyponatremia. Jacob et al. described a similar case in a patient with no risk factors besides aggressive fluid resuscitation after acute bleeding [2]. Benders et al. also reported a case of CPM in a patient with multiple risk factors after the correction of electrolyte disturbances [3].

This case report also emphasizes the need to always be mindfulness with the therapeutic measures we take, even when we think they are innocuous.
